# Sleep and Coping in Early Childhood During the COVID-19 Pandemic

**DOI:** 10.3389/fped.2021.716608

**Published:** 2021-07-30

**Authors:** Sanna Lokhandwala, Jennifer F. Holmes, Gina M. Mason, Christine W. St. Laurent, Cassandra Delvey, Olivia Hanron, Chloe Andre, Katrina Rodheim, Sukhmanjit Kaur, Rebecca M. C. Spencer

**Affiliations:** ^1^Department of Psychological and Brain Sciences, University of Massachusetts Amherst, Amherst, MA, United States; ^2^Developmental Sciences Program, University of Massachusetts Amherst, Amherst, MA, United States; ^3^Neuroscience and Behavior Program, University of Massachusetts Amherst, Amherst, MA, United States; ^4^Institute for Applied Life Sciences, University of Massachusetts Amherst, Amherst, MA, United States

**Keywords:** sleep, coping, early childhood, preschool, COVID-19, stress

## Abstract

Sleep disturbances in early childhood are associated with mood and anxiety disorders. Children also exhibit sleep disruptions, such as nighttime awakenings, nightmares, and difficulties falling asleep, in conjunction with adverse events and stress. Prior studies have examined independently the role of sleep on adaptive processing, as well as the effects of stress on sleep. However, how childhood sleep and children's adaptive behavior (i.e., coping strategies) bidirectionally interact is currently less known. Using a within-subjects design and actigraphy-measured sleep from 16 preschool-aged children (M_age_ = 56.4 months, *SD* = 10.8, range: 36–70 months), this study investigated how prior sleep patterns relate to children's coping during a potentially stressful event, the COVID-19 pandemic, and how prior coping skills may influence children's sleep during the pandemic. Children who woke earlier had greater negative expression both before and during the pandemic. During the pandemic, children slept longer and woke later on average compared to before the pandemic. Additionally, for children engaged in at-home learning, sleeping longer was associated with less negative expression. These findings highlight how sleep behaviors and coping strategies are related, and the stability of this relationship under stress.

## Introduction

The ability to cope with stress is critical to human development. Coping has been defined as the effort to regulate one's emotions, cognition, behavior, physiology, and environment in response to challenging experiences (1, 2). Coping is particularly important in childhood as “regulating under stress” is associated with optimal development of emotional well-being and self-regulation (3). For instance, in children facing a potentially stressful circumstance (i.e., family conflict), children's use of adaptive coping strategies (e.g., problem solving) predicted lower depression 5 months later (4). Likewise, distraction coping (i.e., shifting attention by engaging in another activity) predicted lower anxiety and depression 5 months later (4). Other studies have drawn connections between child coping and later aggression (5), and between early coping skills and later psychiatric difficulties (1). Together, these findings suggest that coping strategies during early life stress may influence longer-term emotional and mental health.

Accumulating evidence suggests that sleep plays a role in children's coping abilities. Preschool-aged children who are nap deprived exhibit more negative emotional responses when faced with a challenging task (i.e., an unsolvable puzzle) and fewer positive emotional responses to a feasible task (i.e., a solvable puzzle) (6). Additionally, the relationship between children's response inhibition and self-regulation may be altered by sleep (7). When children are well-rested, response inhibition is strongly associated with self-regulation (8). However, this association is compromised when children are sleep restricted. Consistent with these experimental findings, higher variability in sleep duration was found to predict unsatisfactory adjustment to preschool (9). Ultimately, disrupted sleep in young children compromises their ability to engage in adaptive emotional responses, and instead prompts them to display negative coping behaviors.

While sleep affects mood and behavior, the relationship is bi-directional, with stress and behavior also affecting sleep [(10); but see also (13), as arousal may be a causal mechanism linking both stress and sleep]. For example, stressors such as peer separation are associated with sleep disturbances such as longer sleep latencies and decreased naptime in toddlers (11). Likewise, acute stress, experienced when a child is separated from their mother during a sibling's birth, resulted in significantly greater negative affect and night awakenings (12). Conversely, higher levels of support-related coping behaviors (i.e., seeking support from others) served as a protective factor against low sleep quality otherwise seen in economically disadvantaged groups (14). These studies suggest both that stressful events experienced by young children may lead to poor sleep, and that certain behavioral coping strategies may also predict better sleep outcomes.

Studies thus far have examined the effects of child sleep on coping, emotion processing, and mood/anxiety, as well as (separately) the role of naturally occurring and experimental stressors on child sleep. It remains unclear how prior sleep patterns may associate with children's coping during a stressful period and, reciprocally, how prior coping skills may influence children's sleep during a stressful time. To begin examining this gap, we utilized actigraphy to objectively track the napping and overnight sleep patterns of young children, both prior to and during a prolonged potentially stressful experience, the SARS-CoV-2 (COVID-19) pandemic. Using parent-report, we also assessed children's coping, to explore how the relations between sleep and coping may vary across time and in the face of changes in stress contexts.

The COVID-19 pandemic provides a naturalistic opportunity to examine how the relations between sleep and coping in early childhood may change when the child is challenged by prolonged stress. In particular, the widespread closing of schools and businesses in the United States, intended to encourage social distancing, brought about unique social and family/peer separation challenges (15, 16). Among adults, the COVID-19 pandemic has been associated with reduced sleep quality and higher rates of passive coping (e.g., escapism, depending on others) during the pandemic than before (17). While studies in children have reported longer sleep times after the pandemic onset relative to before the pandemic (18–20), the pandemic may have resulted in reduced sleep quality and quantity in children (21–23) and naps may be less frequent and shorter (19, 20). However, it is unclear how the effects of sleep and coping may be bidirectionally associated in the context of ongoing stress in young children, particularly considering that coping processes have been suggested to be regulated by situation-related phenomena [e.g., situations that contribute to prolonged and increased stress such as diagnosis of a disease; (24)]. We anticipate the current COVID-19 pandemic to be such a phenomenon given the potential for increased stress for children, associated with factors such as threat of illness and changes in daily routine (e.g., no childcare or limited remote services).

Here, we focused on three forms of coping: *positive coping* (e.g., being hopeful, working with others), *negative coping-emotional expression* (e.g., crying, screaming, blaming others), and *negative coping-emotional inhibition* (e.g., doing nothing, giving up) (2). Further, we examined whether these relations changed across our two timepoints (before and during the COVID-19 pandemic), as well as whether prior sleep associated with current coping and vice versa. We hypothesized that poor sleep quality would be associated with poorer coping strategies (i.e., more negative coping) among children, and that coping would bidirectionally impact child sleep during the pandemic. Alternatively, it may be that the stay-at-home guidelines foster a less strenuous routine in which children exhibit better sleep quality and in turn better coping strategies, independent of prior sleep and coping. In addition, as the pandemic resulted in a number of school and childcare center closures, many children have been participating in remote programs and may vary in the extent of their online learning experience. Participation in at-home or remote learning opportunities, vs. no remote early childhood educational services, may provide some protective effects on sleeping habits (e.g., *via* more structured daily routines) and coping (e.g., *via* maintenance of social connections with peers and teachers). Thus, whether at-home learning opportunities impacted coping and sleep was further explored in *post-hoc* analyses. Understanding these relations will better elucidate how childhood sleep patterns may serve as a marker of coping ability in the face of stressful circumstances.

## Methods and Materials

### Participants

Participants were 19 children aged 36–70 months (M_age_ = 57 months, SD = 10; 5 females) and their caregivers. Participants were recruited from a pool of families in western Massachusetts who 1) had participated in one of our prior studies before the pandemic (within the past 12 months) and 2) had >3 days actigraphy data (25, 26), and 3) had consented to be contacted again for future studies. To be eligible, children were required to have no diagnosis of a sleep disorder or developmental disability, not be using sleep-affecting medications, have normal or corrected-to-normal vision, and have no recent illness or travel across time zones. The caregiver who participated prior to the pandemic was enrolled at the pandemic follow-up.

Three children were excluded due to inadequate actigraphy data (<3 days) at follow-up. Actigraph watches malfunctioned for two children and one child declined to wear the watch. Thus, our final sample, with usable data at both timepoints, included 16 participants (at pandemic follow-up: M_age_= 56.4 months, SD = 10.8, range: 36–70 months; 3 females). The sample was 75% White, 18.8% Black, and 6.3% more than one race, and 18.8% of participants identified as Hispanic. Median reported household income range was $100,000–150,000, which is considered middle to upper income in the United States (27). Prior to the pandemic, approximately 81.4% of respondents worked either full- (62.6%) or part-time (18.8%), with the remaining respondents identifying as students (12.5%) or stay-at-home caregivers (6.3%). During the pandemic, only one respondent (6.3%) reported that they had been entirely laid off; most respondents (56.3%) reported no significant changes in work hours, while the remainder reported reduced work hours either for themselves (12.5%) or their partners (25%).

### Child Sleep Measures

#### Actigraphy

Children wore an Actiwatch Spectrum Plus watch (Philips Respironics, Bend, OR) on their non-dominant wrist for up to 16 consecutive days and nights. The Actiwatch sampled activity at 32 Hz, with a sensitivity of <0.01 g. Activity was stored in 15-s epochs. Children and caregivers were instructed to press an event marker button on the watch to indicate the beginning and end of sleep (naps and overnight) bouts. Caregivers also completed a daily sleep diary for their child, which was used to aid in scoring of actigraphy data. Actigraphy provides a reliable estimate of sleep in children, with 89% agreement when compared to polysomnography (28).

#### Sleep/Wake Diary

Over the 16-day study, caregivers completed a daily sleep diary for their child, recording sleep onset and offset and presence and timing of naps. These records were used to validate scoring of actigraphy data.

### Child Coping and Pandemic Experience Measures

#### Children's Coping Scale – Revised

The Children's Coping Scale - Revised (CCS-R) was used to assess child coping (2). The 29-item questionnaire asks respondents to rate how frequently their child uses various coping strategies when faced with a problem. For each item, possible responses were: “never,” “sometimes,” and “a lot.” The scale was completed twice - once with instructions to consider the child's typical coping over the past year (prior to the pandemic) and again with instructions to consider the child's typical coping over the past 2 weeks.

Three coping dimension scores are derived from this scale: positive coping, negative coping-emotional expression, and negative coping-emotional inhibition. **Positive coping** reflects children's use of active or problem-focused coping strategies, such as “Go out and play and forget about their problem” or “Get a teacher or grown-up to help.” Negative coping-emotional expression (henceforth referred to as **negative expression**) measures a child's tendency to use passive, emotion-focused strategies like “Worry” or “Cry or scream.” Negative coping-emotional inhibition (henceforth referred to as **negative inhibition**) also reflects the tendency to use passive strategies, but with more inhibition of feelings. Example responses include “Give up” or “Keep feelings to self.” The CCS-R has good internal consistency for all coping subscales (positive coping: α = 0.87, negative expression: α = 0.73, negative inhibition: α = 0.66; 2).

#### COVID-19 Child Experience Survey

Caregivers were asked questions about their child's objective experiences with COVID-19 (see Appendix). These questions were taken from the *COVID-19 Adolescent Symptom & Psychological Experience (CASPE) Questionnaire-Parent Version* (29), made publicly available *via* the PhenX Toolkit (30). This survey covered events that adolescents may have experienced during the pandemic, such as school closure or having a family member fall ill. As our goal in this survey was capture whether the younger child had been exposed to specific COVID-19 related events, we used items only from sections A and D of the original CASPE parent survey, streamlining to avoid redundancies (not related to internal consistency) and omitting questions in which parents were asked to guess their children's subjective feelings about COVID-19 and its impacts and those relating to situations specific to older children/adolescents (e.g., references to alcohol, or tobacco). Additionally, we added three questions inquiring about children's average consumption of media related specifically to COVID-19. The full survey is available in the Appendix, and further details regarding the scoring of this survey are available in Data Analysis below.

### Procedure

All procedures were reviewed and approved by the Institutional Review Board at the University of Massachusetts, Amherst. Caregivers consented for their child's and their own participation both prior to and during the pandemic. Data collected prior to the pandemic was part of a larger study assessing the relation between daytime naps and memory in preschool children. Before the pandemic (**T0**), child assent was obtained before fitting the Actiwatch. Concurrently, caregivers were given instructions on how to use the Actiwatch and were provided the sleep diary and in-house demographic questionnaire to complete at any point during the 16-day period. Actiwatches and questionnaires were collected, and caregivers received monetary compensation while children received a storybook. The same procedures took place at the pandemic follow-up (**T1**). However, Actiwatches were delivered by mail and caregivers completed the sleep diary, CCS-R, and the modified CASPE (COVID-19 Child Experience Survey) *via* Qualtrics. T0 took place between July 2019 and February 2020 and T1 took place between May 2020 and early June 2020, during the shutdown of schools and non-essential businesses as part of the state of Massachusetts's stay-at-home advisory (31).

### Data Analysis

#### Child Sleep

Actigraphy data were scored using Actiware software (Philips Respironics, Bend, OR), with the proprietary algorithm set at medium sensitivity (32), and standard scoring procedures (33). To confirm sleep intervals, sleep diaries and event markers were used. If a discrepancy of >15 min was found between sources, consensus was sought between trained researchers. Participants' data was excluded and not scored if the actigraphy record displayed <3 nights of clearly identifiable sleep bouts.

**Figure 1:**
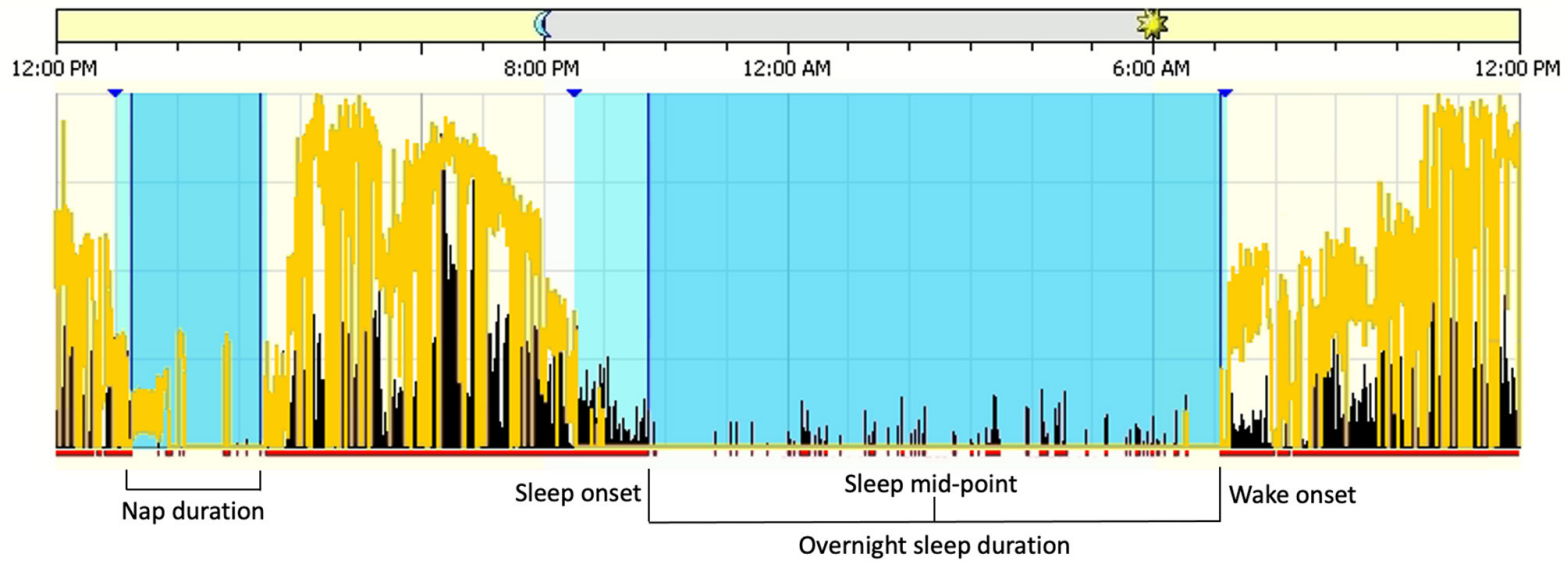
Sleep measures derived from the scored Actigraphy files. Blue sections denote sleep intervals and lighter blue sections denote rest intervals. The yellow lines are a measure of white light, or luminance, and black lines are activity counts per 15-s epoch.

Sleep measures were derived from the scored actigraphy records (see Figure 1). *Sleep onset* was defined as the first 3 min of consecutive sleep epochs. *Wake onset* was defined as the last 5 min of consecutive sleep epochs. *Sleep mid-point* was determined as the absolute mid-point between sleep onset and wake onset. *Overnight sleep duration* and *nap duration* were determined as the intervals between sleep onset and wake onset. Total *24-h sleep duration* was the sum of nap duration plus the subsequent overnight sleep duration. *Sleep efficiency* was determined by dividing overnight sleep duration by the total time in bed. Actigraphy data were averaged across all usable days for each participant at each time point.

Prior to the pandemic, children had, on average, 12 days of usable actigraphy data (M = 12.3, SD = 3.6, range: 3–16), including 8.7 (SD = 2.7) weekdays and 3.4 (SD = 0.9) weekend days. At follow-up, children had 11 days of usable actigraphy data on average (M = 11.3, SD = 4.5, range: 3–16 days), including 8.1 (SD = 3.2) weekdays and 3.3 weekend days (SD = 1.3). Number of usable days in actigraphy records was not correlated with sleep measures at T0 (*p*s > 0.08) nor T1 (*p*s > 0.40). At T0, sleep timing measures differed between weekdays and weekends, such that sleep onset, sleep mid-point, and wake onset were significantly later on weekends (*p*s < 0.04). At T1, there were no differences between weekday and weekend sleep measures. Lastly, the ratio of usable weekdays and weekend days were similar between T0 and T1 (*p* = 0.68).

#### Child Emotion and Adaptive Behavior

CCS-R items were scored on a 3-point scale. Coping scores were determined as the average of responses on items designated to exemplify each dimension (2). Each coping dimension (positive coping, negative expression, and negative inhibition) had a possible score range of 0–2, with higher scores representing greater use of such coping strategies.

We created a scoring rubric for the *COVID-19 Child Experience Survey* (see Table 1; full rubric available in Appendix), as no rubric or psychometric data had been available for the original CASPE parent survey at the time of our study. Nonetheless, our scoring approach was similar to that of other recent studies making use of CASPE questionnaire items (34, 35). Item scores were weighted more heavily depending on the severity of the event or experience, as determined *via* discussion and consensus from all authors. Positive experiences (i.e., protective factors) or lack of negative events were scored as “0.” Items were considered individually and as a composite score. This composite score had a possible range from 0 to 18, with higher scores representing more negative events or experiences in the child's life related to the pandemic.

**Table 1:** Sample of questions and corresponding scores used for the child experience survey.

**COVID-19 child experience survey questions and scoring rubric**		
**Question**	**Response options**	**Score**
Has your child been tested for COVID-19?	Yes	1
	No	0
If yes, was the COVID-19 test positive?	Yes	1
	No	0
Has anyone in your child's household or extended family been hospitalized because they had COVID-19?	Yes	1
	No	0
Following school closures, how did your child continue with schoolwork?	School sent printed packets	0
	School sent online assignments	0
	School organized online classes	0
	Signed up for different academic program	0
	Child was already homeschooled	0
	There has been no school since then	1
How often is your child getting outside of your house for allowed stay-at-home advisory activities?	Multiple times a day	0
	Once a day	0
	Every couple of days	1
	Once a week	1
	Less than once a week	2
How informed do you think your child is about COVID-19?	Not at all	1
	A little bit	0
	Somewhat	0
	Quite a bit	0
	Extremely	1

*Full list of questions and scoring rubric can be found in Appendix*.

#### Statistical Analyses

Statistical analyses were run using SPSS (Version 27.0). For normally distributed sleep measures [overnight sleep duration, 24-h sleep duration, wake after sleep onset (WASO)], a repeated measures MANOVA was used with time of assessment (T0 vs. T1) as a within-subjects factor. For non-normally distributed sleep measures (nap duration, sleep onset, sleep mid-point, wake onset, sleep onset latency, sleep efficiency), Wilcoxon signed-rank tests were used to assess changes in sleep from T0 to T1. A second repeated measures MANOVA was used to compare CCS-R measures from T0 to T1 (where T0 was retrospective for CCS-R) with time of assessment as a within-subjects factor. Pearson's and Spearman's correlations (for non-normally distributed variables) were used to determine associations between sleep and coping measures.

Pearson's and Spearman's correlations were also used to characterize the relationship between the composite COVID-19 Child Experience survey scores and sleep measures, to explore whether children's exposure to pandemic-related events modulated any associations between sleep and coping. *Post-hoc*, we ran two-tailed independent samples *t*-tests (for normally distributed data) and Mann-Whitney U tests (for non-normally distributed data) to examine sleep differences between children who were participating in at-home learning and those who were not.

## Results

### Sleep and Coping Behavior at Baseline and Early Pandemic

First, we assessed whether sleep duration, quality, and timing differed during the pandemic (T1) relative to baseline (T0; Table 2). Average duration between testing timepoints was 197.9 days (SD = 80.1, range: 79–313). There was a main effect of time of assessment on sleep measures [*F*_(3, 13)_ = 6.54, *p* = 0.006; Wilks' Λ = 0.40, partial η^2^ = 0.60]. Overnight sleep duration was significantly longer during the pandemic relative to baseline [*F*_(1, 15)_ = 10.20, *p* = 0.006, partial η^2^ = 0.41], with children sleeping 27.5 min longer on average. Total 24-h sleep duration did not significantly differ from T0 to T1 [*F*_(1, 15)_ = 0.24, *p* = 0.63]. Nap duration also did not differ from T0 to T1 (*Z* = −1.21, *p* = 0.23), though only 5 children were still napping at T1 (vs. 12 at T0). Overnight sleep efficiency, a common measure of sleep quality, remained consistent from T0 to T1 (*Z* = −0.91, *p* = 0.37*)*. In terms of sleep timing, sleep onset did not significantly differ from T0 to T1 (Z = −1.08, *p* = 0.29), but sleep mid-point and wake onset were significantly later by 32 and 46 min, respectively, at T1 (*Sleep mid-point*: *Z* = −3.03, *p* = 0.002; *Wake onset:* Z = −3.16, *p* = 0.002).

**Table 2:** Sleep and coping behavior before (T0) and during (T1) pandemic.

	**T0**	**T1**	
	**M (SD)**	**M (SD)**	***p***
**Sleep**			
Overnight sleep duration (min)	594.8 (37.1)	622.3 (36.4)	0.006^b^
Nap duration (min)	80.5 (20.8)	70.8 (32.4)^a^	0.225
Total 24-h sleep duration (min)	628.6 (29.4)	631.6 (30.7)	0.631
Sleep onset	8:53 p.m. (56.4 min)	9:10 p.m. (93.6 min)	0.281
Sleep mid-point	1:51 a.m. (50.4 min)	2:24 a.m. (85.8 min)	0.002^b^
Wake onset	6:49 a.m. (51 min)	7:35 a.m. (84.6 min)	0.002^b^
Sleep onset latency (min)	23.4 (12.8)	25.7 (17.5)	0.796
Wake after sleep onset (WASO; min)	58.4 (20.4)	60.1 (15.1)	0.596
Sleep efficiency (%)	84.9 (4.6)	85.4 (4.5)	0.365
Nap frequency (naps/week)	2.9 (2.3)	0.8 (1.8)	0.002^b^
**Coping**			
Positive coping	1.4 (0.6)	1.2 (0.5)	0.009^b^
Negative coping – emotional expression	0.5 (0.4)	0.6 (0.4)	0.026^b^
Negative coping – emotional inhibition	0.4 (0.4)	0.4 (0.4)	0.751

a *n = 5;*

b *p < 0.05*.

Baseline measures of overnight sleep duration, nap duration, sleep mid-point, and wake onset were not associated with age (*p*'s > 0.82). Sleep duration and timing measures during the pandemic were also not predicted by age (*p*'s > 0.54). Nap length at T1 was marginally associated with child age (*r* = −0.86, *p* = 0.06).

Next, we explored how children's coping strategies changed from T0 to T1 (Table 2). CCS-R scores were consistent with those of previous studies in this age range (positive coping: 1.03–1.20, negative coping: 0.53–0.69, negative inhibition: 0.39–0.56) (2). There was a main effect of time of assessment on CCS-R scores [*F*_(3, 13)_ = 6.29, *p* = 0.007; Wilks' Λ = 0.41, partial η^2^ = 0.60]. Caregivers reported significantly lower child positive coping during the pandemic than before [*F*_(1, 15)_ = 9.06, *p* = 0.009, partial η^2^ = 0.38]. Child negative expression was also higher during the pandemic compared to prior [*F*_(1, 15)_ = 6.14, *p* = 0.026, partial η^2^ = 0.29], yet individual differences were preserved as negative expression scores at T0 and T1 were strongly correlated (*r* = 0.90, *p* < 0.001). Negative inhibition scores did not change from T0 to T1 (*p* = 1.00).

### Child COVID-19 Experience

COVID-19 Child Experience survey composite scores ranged from 2 to 6 with a mean of 3.1 (SD = 0.96). Frequency distributions for the composite score, and for each individual question, are available in the Supplementary Table 1 and Supplementary Figure 1. Overall, no parents reported that their child had been tested for or contracted COVID-19, nor had any of their household members been quarantined due to possible exposure at the time of our follow-up. One parent reported a child losing an extended family member due to COVID-19. Two children had friends who had tested positive for COVID-19. All parents reported that their household began following stay-at-home orders and social distancing guidelines in mid-March, 2020. During this period, most parents (81.3%) reported that their child spent time outdoors or being active “once” or “multiple times” per day, while 18.8% of parents reported engaging in such activities “once a week” to “every couple of days.” All children in our cohort experienced school closures early in the pandemic.

### Associations Between Sleep and Coping

**Figure 2:**
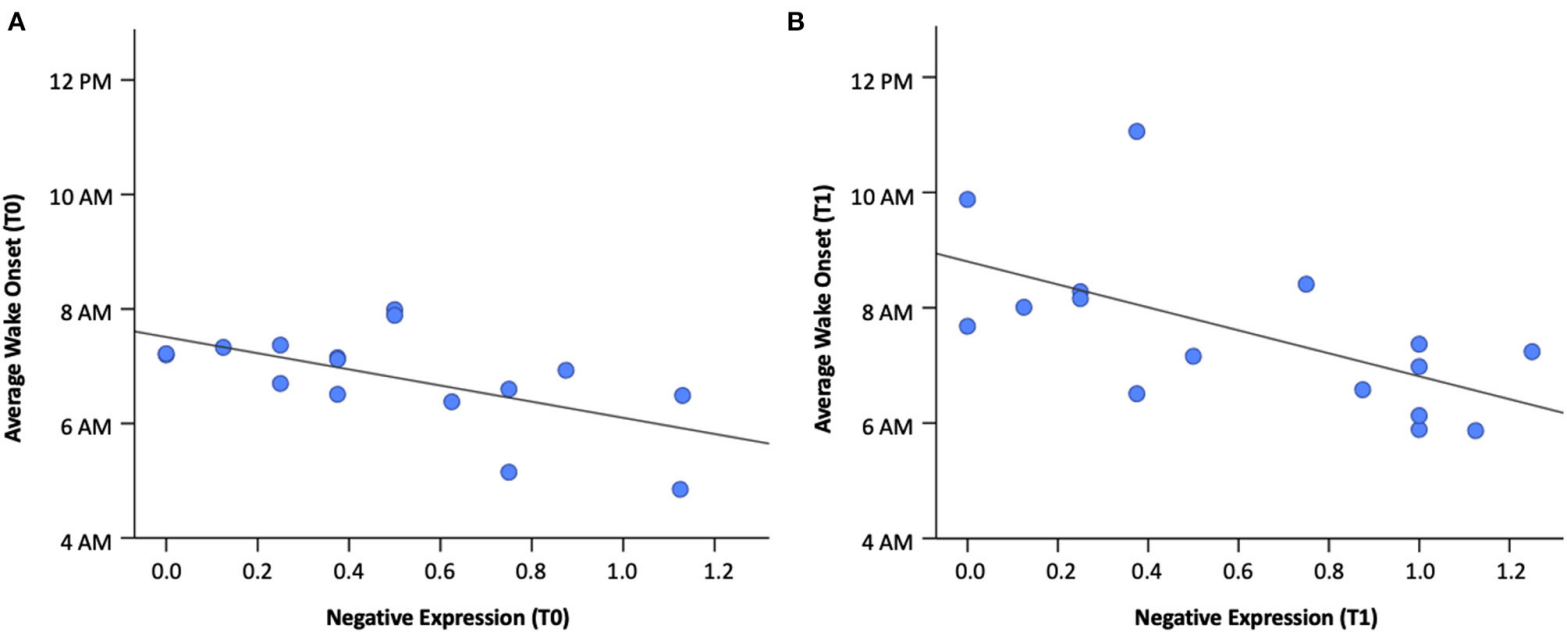
Relation between wake onset and negative expression **(A)** at baseline (T0) and **(B)** mid-pandemic (T1).

Correlations between sleep and coping dimensions at T0 and T1 are shown in Table 3 and Supplementary Table 2. No significant associations were found between positive coping and sleep measures at either time point. Negative expression at T0 was negatively associated with wake onset at T0 (*r*_*s*_= −0.56, *p* = 0.02), suggesting that children who woke up earlier displayed greater negative expression-type coping strategies (see Figure 2A). A comparable relationship also existed at T1 (*r*_*s*_ = −0.70, *p* = 0.003; see Figure 2B). Wake onset, at both T0 and T1, did not correlate with sleep duration (T0: *p* = 0.62; T1: *p* = 0.88), suggesting that the relationship with negative expression was not due to changes in sleep length, but rather shifted sleep timing.

**Table 3:** Correlations between sleep measures of interest and coping subscales (*N* = 16).

**Variables**	**1**	**2**	**3**	**4**	**5**	**6**	**7**	**8**	**9**	**10**	**11**
**Baseline (T0)**											
1. Overnight sleep duration	–										
2. Sleep onset	−0.47	–									
3. Sleep mid-point	−0.16	0.95^d^	–								
4. Wake onset	0.13	0.71^c^	0.87^d^	–							
5. Positive coping	0.29	−0.11	0.25	−0.06	–						
6. Negative coping – emotional expression	−0.49^a^	−0.21	−0.43	−0.56^b^	−0.34	–					
**Mid-Pandemic (T1)**											
7. Overnight sleep duration	0.56^b^	−0.26	−0.09	0.26	−0.27	−0.29	–				
8. Sleep onset	−0.17	0.83^d^	0.84^d^	0.79^c^	0.06	−0.45	−0.21	–			
9. Sleep mid-point	−0.04	0.81^d^	0.93^d^	0.92^d^	−0.15	−0.47	0.12	0.87^d^	–		
10. Wake onset	0.01	0.77^c^	0.90^d^	0.93^d^	−0.14	−0.49^a^	0.26	0.79^d^	0.97^d^	–	
11. Positive coping	0.21	0.15	0.25	0.08	0.93^d^	−0.44	−0.31	0.26	0.02	−0.02	–
12. Negative coping – emotional expression	−0.31	−0.36	−0.51^b^	−0.70^c^	−0.06	0.90^d^	−0.21	−0.61^b^	−0.68^c^	−0.70^c^	−0.18

a *p < 0.06;*

b *p < 0.05;*

c *p < 0.01;*

d *p < 0.001*.

**Figure 3:**
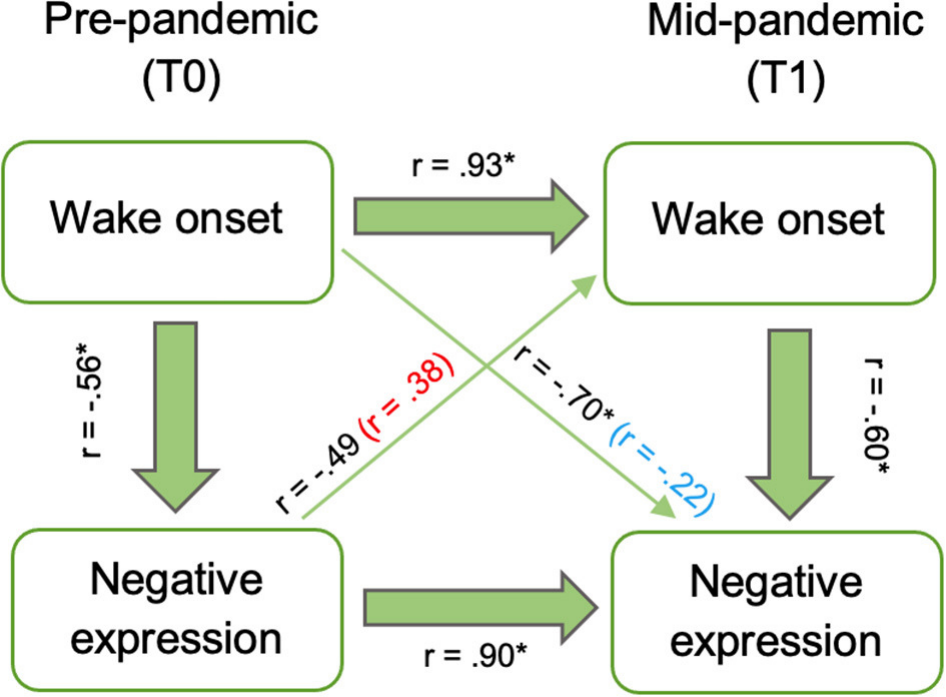
Relations between baseline (T0) wake onset and negative expression and mid-pandemic (T1) wake onset and negative expression. Associations after controlling for T1 negative expression (red) and T1 wake onset (blue) included in parenthesis. Asterisks indicate significant correlations at *p* < 0.05.

We considered whether coping prior to the pandemic was independently associated with sleep during the pandemic. Negative expression at T0 marginally related to wake onset at T1 (*r*_*s*_ = −0.49, *p* = 0.053), however this was driven by the strong correlation between negative expression at T0 and T1, as the relation between T0 negative expression and T1 wake onset became non-significant when controlling for T1 negative expression (*r*_*s*_ = 0.38, *p* = 0.12). Wake onset at T0 was associated with negative expression at T1 (*r*_*s*_ = −0.70, *p* = 0.002), but this was driven by the strong correlation between wake onset at T0 and T1 (*r*_*s*_ = 0.93, *p* < 0.001) and became non-significant when controlling for wake onset at T1 (*r*_*s*_ = −0.22, *p* = 0.44; Figure 3).

### COVID-19 Impact and Sleep

We assessed whether changes in children's sleep related to their pandemic experiences using the Child Experience survey composite score. This score did not correlate with sleep duration, sleep mid-point, or wake onset at T1 (*p*'s > 0.15).

Fifteen children were enrolled in preschool at both T0 and T1, while one child was not enrolled in preschool at either time point but was homeschooled. For those in school, nearly all children experienced school closures beginning in mid-March 2020. The average time from school closure to testing (T1) was 61 days (SD = 13.1). Time from school closure to T1 testing negatively correlated with total 24-h sleep (*r* = −0.52, *p* = 0.048) suggesting perhaps that sleep duration decreased as lockdowns were less acute. No other sleep or coping measures correlated with time of assessment in relation to the start of the stay-at-home order. Half of parents (*n* = 8) reported that their child was not receiving any virtual learning or at-home instruction at T1, despite time of assessment being within the traditional school year, while the other half were receiving at-home instruction in the form of online academic programs, school-provided packets and/or recommendations, or parent-initiated learning opportunities. We explored whether their sleep patterns differed based on at-home learning instruction status (Table 4). Children receiving at-home instruction slept 44 min longer overnight than those who were not receiving at-home instruction [*t*_(14)_ = 2.92, *p* = 0.01, *d* = 1.46]. Sleep mid-point (*U* = 26.0, *p* = 0.53) and wake onset differences between groups were both non-significant (*U* = 30.0, *p* = 0.83). Sleep onset, while remaining fairly constant from T0 to T1, did marginally differ between these groups (*U* = 15.0, *p* = 0.07). Children receiving at-home learning went to bed 85 min earlier, on average, compared to those not currently receiving at-home instruction (*M* = 8:23 p.m. vs. *M* = 9:48 p.m.). These groups did not differ by age [*t*_(14)_ = −0.25, *p* = 0.81] nor in reported household income (*U* = 23.0, *p* = 0.33).

**Table 4:** Sleep and coping between learning engagement status groups at T1.

	**At-home instruction (*n* = 8)**	**No at-home instruction (*n* = 8)**	
	**M (SD)**	**M (SD)**	***p***
Age	55.8 (11.6)	57.1 (10.7)	0.809
**Sleep**			
Overnight sleep duration (min)	643.1 (27.2)	600.6 (32.1)	0.011^b^
Sleep onset	8:23 p.m. (41.9 min)	9:48 p.m. (115.8 min)	0.073
Sleep mid-point	1:42 a.m. (46.8 min)	2:47 a.m. (115.8 min)	0.529
Wake onset	7:04 a.m. (54.7 min)	8:05 a.m. (112.2 min)	0.834
**Coping**			
Positive coping	0.88 (0.6)	1.40 (0.3)	0.052^a^
Negative coping – emotional expression	0.52 (0.4)	0.63 (0.4)	0.597
Negative coping – emotional inhibition	0.23 (0.3)	0.31 (0.2)	0.737

a *p < 0.06;*

b *p < 0.05*.

Positive coping scores at T1 differed between those receiving at-home instruction and those who were not (see Table 4). For the at-home learners, positive coping was marginally lower [*t*_(14)_ = −2.13, *p* = 0.052, *d* = −1.06]. Negative expression was also lower for those receiving at-home instruction, but this difference was not significant [*t*_(14)_ = −0.54, *p* = 0.60, *d* = −0.27].

Due to differences in sleep durations and bedtimes between children receiving at-home instruction vs. not, we examined associations between these sleep measures and positive coping and negative expression scores (at T1) within groups. Positive coping was not associated with sleep duration or sleep onset for either group (*p'*s > 0.36). Sleep duration was negatively associated with negative expression scores in children receiving at-home instruction (*r* = −0.69, *p* = 0.047), but not in children who were not receiving at-home instruction (*r* = 0.25, *p* = 0.55). That is, for remote learners, longer sleep durations were related to lower negative expression. Sleep onset was also associated with negative expression in children who were receiving at-home learning instruction (*r*_*s*_ = −0.93, *p* = 0.001) and in children not receiving at-home instruction (*r*_*s*_ = −0.77, *p* = 0.02). In both groups, later sleep onset was related to less negative expression.

## Discussion

In the present study, we examined how prolonged stress may influence young children's coping strategies and sleep behavior, and how prior sleep and coping affect children's sleep and coping during periods of stress. Sleep and coping strategies were considered at two timepoints: prior to and during the COVID-19 pandemic. Overall, the pandemic brought about unique changes for sleep, as children slept longer (particularly if they were engaged in at-home learning) and woke up later during this distinctively stressful period. Further, children exhibited greater negative expression during the pandemic than before. Children's negative expression was associated with wake onset, such that earlier wake times were associated with greater negative expression both before and during the pandemic. These findings suggest that although the pandemic was associated with changes in children's sleep and coping strategies independently, the bidirectional associations between sleep and coping observed under typical circumstances were preserved across the pandemic.

Given our findings, it is necessary to consider how wake times may be important in relation to coping. Chronotype [i.e., one's preference for waking up and going to bed earlier or later (36)] shows a range of natural variation across individuals and has been linked to mood disruption, although primarily in adult studies (37–41). Whereas, most evidence has demonstrated that a later chronotype is associated with mood disruptions such as depressive symptoms (38), some prior work suggests that earlier wake onsets may be indicative of heightened mood disruption (39). Early wake onsets and other circadian anomalies may indicate a misalignment between sleep and one's circadian clock relating to losses in synchronization to environmental 24-h rhythms, changes in sleep architecture, and desynchronization internally among body clocks (40). This is associated with a neurohormonal stress response that contributes to increased negative emotionality (39, 41). This may also be why wake time was associated with negative expression and not other sleep timing features like mid-sleep point and sleep onset. Nonetheless, the fact that we did not find a direct relation between children's sleep durations and positive coping, in particular, contradicts studies in adolescents that suggest associations between sleep length and coping strategies. For example, work done in 256 adolescents found that when adolescents slept longer, they engaged in greater active coping [e.g., problem solving and seeking peer support, (41)]. It is possible that we did not find this relation between sleep duration and positive coping due to small sample size and insufficient variability in sleep duration among our participants. However, sleep duration and negative expression were related in children receiving at-home instruction. This contradiction between sleep and positive coping (i.e., active coping) may be due to age-related differences in coping strategies (3), developmental changes in sleep and circadian rhythms (42), or both. Thus, it is important to extend this research into examining across development and age ranges. Nevertheless, the fact that the association between wake onset and negative expression was maintained both prior to and during the pandemic suggests that within participants, relations between sleep (specifically, children's circadian rhythm) and coping may be conserved during both normative times and times of stress.

One potential pathway relating sleep and coping in early childhood is through mood and behavior. Insufficient sleep in young children has been associated with indicators of poor mood and behavior such as depressive symptoms and anxiety (43). For instance, parent reports of preschool children's sleep onset latency and refusal to sleep alone independently predict depression and anxiety symptom severity across time (44). Likewise, reductions in sleep duration and quality (e.g., trouble falling asleep and oversleeping) at 8 years have been shown to predict greater anxiety, depression, and internalizing/externalizing behaviors by age 10 (45). These results are important given that depression specifically has been linked with more avoidance coping, possibly indicating both direct and indirect connections between sleep, coping skills, and mood-related difficulties (46).

In a *post-hoc* analysis, we examined how sleep differed between children who participated in at-home learning after school closures vs. children who did not. Children engaged in some form of at-home learning slept significantly longer during the night than children not engaged in at-home learning. While prior studies have found longer sleep duration in young children during the pandemic (18–20), our findings elucidate that at-home learning might play a role in children's sleep length. The protective effect of at-home learning may be related to exposure to a more structured daytime schedule which in turn may have contributed to more sleep regularity and more consistent sleep routines, compared to the potential lack of obligations that children without at-home learning faced. Future studies should also examine whether at-home learning schedules are more stimulating and taxing on average than not having an at-home learning routine, and investigate how these learning schedules may change sleep parameters. Additionally, although at-home learning may take different forms, this may involve increased electronic screen time which could increase light exposure. Given that light influences circadian rhythms (47–49), future work might examine the role of light exposure on sleep variables. Teasing apart how much light exposure, at what time of the day, and from what sort of devices/environments may be helpful in elucidating how sleep is impacted in remote and non-remote learners. In examining the relationship between sleep and coping in children who engaged in at-home learning and those who did not, we found that for remote learners, longer sleep duration was associated with lower scores of negative expression. Future research should determine if children without adequate sleep are unable to appropriately cope with the unique challenges that come with online learning, and how this may be associated with varying daytime structure and stimulating activities.

The present study has several limitations. First, the sample size was small and composed primarily of high SES families. It is possible that these caregivers had resources to mitigate the challenges brought on by the pandemic, potentially buffering relations between child sleep and pandemic experience and therefore our findings may not be generalizable to other populations. Second, our state-based coping measure, the CCS-R, was filled out by parents currently and retrospectively. As such, parent bias may have led to inaccurate reporting. More so, retrospective reporting of T0 at T1 is imperfect. Further, the CCS-R did not explicitly ask parents to think of their child's coping strategies before the pandemic. Thus, recency effects may have affected parents' ratings and may not be reflective of true pre-pandemic coping baseline. Additionally, early childhood is emblematic of developmental changes in sleep where children transition from taking regular naps in their day to consolidating sleep to overnight (50–52). A number of children in our sample were not napping by T1. Thus, it is difficult to suggest whether the cessation of napping is due to normative developmental change or whether it is symptomatic of the pandemic. Further, it is possible our analyses are limited by the assumption that the relationship between sleep and coping is linear. However, it may be that the relationship is non-linear, an association we did not examine due to our small sample size. Also, other potential factors have not been examined in this paper that may be moderators of this relationship between sleep and coping (e.g., light exposure, physical activity). Finally, the impact of the pandemic was relatively low in this sample, which again may limit generalizability of our results to families with similar pandemic related stress levels. For example, we found that children's pandemic experiences did not relate with sleep measures. This may be due to low variability in responses as most parents did not report negative experiences beyond school closures or less time spent outdoors/engaged in physical activity.

Strengths of this study include the use of actigraphy, which permitted us to measure children's objective sleep compared to relying on parent report measures. Further, the study design allowed us to assess changes in sleep and coping within the same children, alleviating the concern of individual differences distorting results. While our sample size is small, the associations presented here may serve as groundwork for larger longitudinal studies that test the relationships between sleep and coping during periods of stress.

Together, these data suggest that children's sleep and coping strategies are related, and that this relation during typical times may be indicative of sleep and coping relations during times of stress. Specifically, our work highlights that sleep timing (i.e., wake onset) and duration may be markers of coping abilities during early childhood. In order to inform future interventions that promote healthy emotional development, additional research is needed to replicate and expand on the current findings and to determine how wake onset and coping are mechanistically related.

## Data Availability Statement

The raw data supporting the conclusions of this article will be made available by the authors, without undue reservation.

## Ethics Statement

The studies involving human participants were reviewed and approved by Institutional Review Board at the University of Massachusetts, Amherst. Written informed consent to participate in this study was provided by the participants' legal guardian/next of kin.

## Author Contributions

RS, JH, GM, and OH contributed to the conception and design of the study. SL, JH, GM, CL, CD, OH, CA, KR, and SK contributed to data collection and organization. SL and JH performed the statistical analyses and wrote the first draft of the manuscript. All authors contributed to manuscript revision, read, and approved the submitted version.

## Conflict of Interest

The authors declare that the research was conducted in the absence of any commercial or financial relationships that could be construed as a potential conflict of interest.

## Publisher's Note

All claims expressed in this article are solely those of the authors and do not necessarily represent those of their affiliated organizations, or those of the publisher, the editors and the reviewers. Any product that may be evaluated in this article, or claim that may be made by its manufacturer, is not guaranteed or endorsed by the publisher.
