# Supplementary material for: Sleep and Coping in Early Childhood During the COVID-19 Pandemic
Source: Front Pediatr. 2021 Jul 30;9:716608. doi: 10.3389/fped.2021.716608 (PMC8360857; doi:10.3389/fped.2021.716608)
Supplement: Supplementary file 1 [file Data_Sheet_1.pdf]

APPENDIX.

Supplemental Table S1. COVID-19 Child Experience Survey and Sample Distributions for Each Question

Question	Response Options	Score	Response (% of Sample)
1. Has your child been tested for COVID-19?	Yes	1	0%
	No	0	100%
1a. If yes, was the COVID-19 test positive?	Yes	1	n/a
	No	0	n/a
2. Has your child been instructed by a doctor to quarantine at home due to COVID-19?	Yes	2	0%
	No	0	100%
3. How many people in your household have or have had COVID-19?	0-1	0	100%
	2	1	0%
	>2	2	0%
4. Has anyone in your child's household or extended family been hospitalized because they had COVID-19?	Yes	1	6%
	No	0	94%
5. Has anyone in your child's household or extended family died because they had COVID-19?	Yes (household)	4	0%
	Yes (extended family)	2	6%
	No	0	94%
6. Have any of your child's friends (or friend's family members) had COVID-19?	Yes	1	12.5%
	No	0	87.5%
7. On what date did you begin to have your child stay at home from school and other activities (i.e. social distancing) because of the COVID-19 outbreak?	Any date	1	100%
	School did not close	0	0%
8. Following school closures, how did your child continue with schoolwork?	School sent printed packets	0	6%
	School sent online assignments	0	6%
	School organized online classes	0	0
	Signed up for different academic program	0	12.5%
	Child was already homeschooled	0	6%
	Other (please specify)	0	19%
	There has been no school since then	1	50%
9. How often is your child getting outside of your house for allowed stay-at-home advisory activities?	Multiple times a day	0	50%
	Once a day	0	31%
	Every couple of days	1	19%
	Once a week	1	0%

	Less than once a week	2	0%
10. Since your child's school has closed, how often does he/she talk with friends online?	Every day or almost every day	0	0%
	Several times a week	0	6%
	About once a week	1	31%
	Less often	1	62.5%
11. How informed do you think your child is about COVID-19?	Not at all	1	12.5%
	A little bit	0	56%
	Somewhat	0	12.5%
	Quite a bit	0	19%
	Extremely	1	0%
12. Please indicate your child's average weekly consumption of adult-targeted news related to COVID-19 (in hours)			
13. Please indicate your child's average weekly consumption of child-friendly media related to COVID-19 (in hours)	Sum of Q.12 and Q.13:		
	0-7 hours	0	94%
	>7 hours	1	6%
	Total Score Range	0-18	

Supplemental Figure S1. Distribution of CASPE composite scores across child participants.

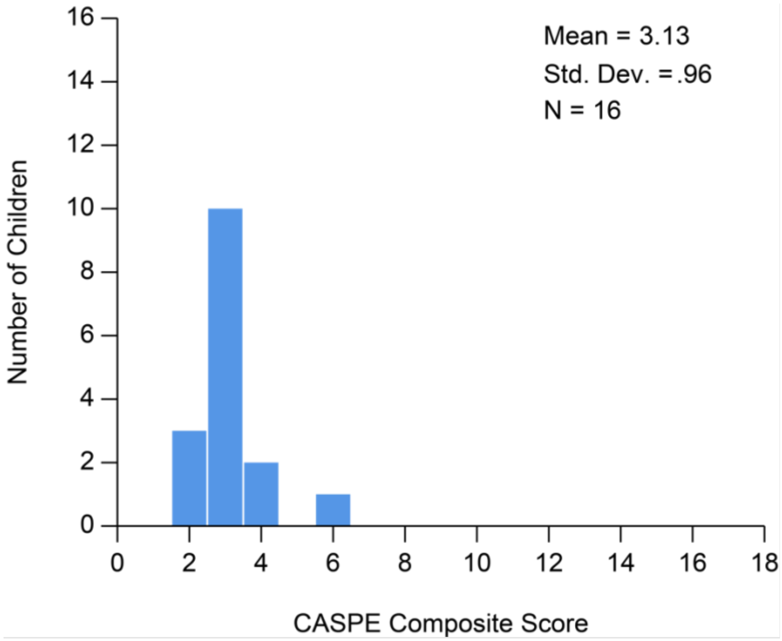

Supplemental Table S2. Correlations Between Standard Deviations (SD) of Sleep Measures and Coping Subscales (*N* = 16)

Variables	1	2	3	4	5	6	7	8	9	10	11
**Baseline (T0)**											
1. Overnight sleep duration SD	-										
2. Sleep onset SD	.67[b]	-									
3. Sleep mid-point SD	.72[b]	.84[b]	-								
4. Wake onset SD	.72[b]	.19	.61[a]	-							
5. Positive coping	-.13	-.08	-.19	-.24	-						
6. Negative expression	.01	.26	.45	.35	-.34	-					
**Mid-Pandemic (T1)**											
7. Overnight sleep duration SD	.17	-.09	.18	.43	-.44	-.08	-				
8. Sleep onset SD	.19	.19	.13	.19	-.31	-.06	.44	-			
9. Sleep mid-point SD	.33	.52[a]	.34	.19	-.12	-.08	.43	.59[a]	-		
10. Wake onset SD	.57[a]	.38	.27	.36	-.09	-.26	.84[c]	.36	.72[b]	-	
11. Positive coping	.07	.01	-.06	-.27	.93[c]	-.44	-.30	-.23	.03	.02	-
12. Negative expression	.16	.40	.52[a]	.29	-.06	.90[c]	-.21	-.07	-.09	-.31	-.18

[a]$p < .05$. [b]$p < .01$. [c]$p < .001$.
